# Supplementary material for: Modeling glioblastoma heterogeneity as a dynamic network of cell states
Source: Mol Syst Biol. 2021 Sep 16;17(9):e10105. doi: 10.15252/msb.202010105 (PMC8444284; doi:10.15252/msb.202010105)
Supplement: Supplementary file 6 — Source Data for Figure 5 [file MSB-17-e10105-s004.zip › Figure5A_sourcedata/GSEA_3071/nosigdb_stateI.GseaPreranked.1622795145664/SEURAT_CELLCYCLE.html]

Details for gene set SEURAT\_CELLCYCLE[GSEA]

|  || Dataset | state13071 |
| Phenotype | NoPhenotypeAvailable |
| Upregulated in class | na\_neg |
| GeneSet | SEURAT\_CELLCYCLE |
| Enrichment Score (ES) | -0.6061098 |
| Normalized Enrichment Score (NES) | -3.0472414 |
| Nominal p-value | 0.0 |
| FDR q-value | 0.0 |
| FWER p-Value | 0.0 |
Table: GSEA Results Summary

  

Fig 1: Enrichment plot: SEURAT\_CELLCYCLE      
 Profile of the Running ES Score & Positions of GeneSet Members on the Rank Ordered List

  

| PROBE | GENE SYMBOL | GENE\_TITLE | RANK IN GENE LIST | RANK METRIC SCORE | RUNNING ES | CORE ENRICHMENT || 1 | MSH2 |  |  | 1438 | -0.252 | -0.4144 | No |
| 2 | CTCF |  |  | 1450 | -0.253 | -0.4127 | No |
| 3 | CHAF1B |  |  | 1554 | -0.262 | -0.4376 | No |
| 4 | TTK |  |  | 1620 | -0.270 | -0.4513 | No |
| 5 | KIF2C |  |  | 1735 | -0.284 | -0.4790 | No |
| 6 | CCNB2 |  |  | 1757 | -0.288 | -0.4794 | No |
| 7 | RAD51 |  |  | 1845 | -0.299 | -0.4990 | No |
| 8 | CASP8AP2 |  |  | 1857 | -0.300 | -0.4963 | No |
| 9 | TIPIN |  |  | 1942 | -0.312 | -0.5147 | No |
| 10 | RANGAP1 |  |  | 2207 | -0.354 | -0.5848 | No |
| 11 | CENPA |  |  | 2220 | -0.357 | -0.5813 | No |
| 12 | TUBB4B |  |  | 2291 | -0.370 | -0.5944 | No |
| 13 | CKS1B |  |  | 2332 | -0.380 | -0.5987 | Yes |
| 14 | MCM2 |  |  | 2349 | -0.383 | -0.5958 | Yes |
| 15 | CKAP2 |  |  | 2357 | -0.385 | -0.5903 | Yes |
| 16 | POLA1 |  |  | 2376 | -0.388 | -0.5880 | Yes |
| 17 | UHRF1 |  |  | 2404 | -0.393 | -0.5882 | Yes |
| 18 | BUB1 |  |  | 2416 | -0.396 | -0.5836 | Yes |
| 19 | CKS2 |  |  | 2466 | -0.404 | -0.5900 | Yes |
| 20 | AURKA |  |  | 2467 | -0.404 | -0.5821 | Yes |
| 21 | BLM |  |  | 2469 | -0.404 | -0.5745 | Yes |
| 22 | KIF11 |  |  | 2488 | -0.410 | -0.5717 | Yes |
| 23 | CDC20 |  |  | 2540 | -0.425 | -0.5783 | Yes |
| 24 | NCAPD2 |  |  | 2563 | -0.431 | -0.5762 | Yes |
| 25 | UBR7 |  |  | 2611 | -0.442 | -0.5813 | Yes |
| 26 | CDCA8 |  |  | 2695 | -0.470 | -0.5963 | Yes |
| 27 | MCM6 |  |  | 2699 | -0.471 | -0.5879 | Yes |
| 28 | ANLN |  |  | 2702 | -0.472 | -0.5793 | Yes |
| 29 | NDC80 |  |  | 2723 | -0.478 | -0.5758 | Yes |
| 30 | EXO1 |  |  | 2728 | -0.479 | -0.5676 | Yes |
| 31 | BRIP1 |  |  | 2739 | -0.482 | -0.5610 | Yes |
| 32 | UNG |  |  | 2758 | -0.489 | -0.5567 | Yes |
| 33 | CENPF |  |  | 2761 | -0.490 | -0.5477 | Yes |
| 34 | NUF2 |  |  | 2781 | -0.497 | -0.5435 | Yes |
| 35 | SLBP |  |  | 2792 | -0.499 | -0.5367 | Yes |
| 36 | WDR76 |  |  | 2800 | -0.501 | -0.5289 | Yes |
| 37 | DSCC1 |  |  | 2828 | -0.512 | -0.5267 | Yes |
| 38 | CDCA3 |  |  | 2829 | -0.513 | -0.5167 | Yes |
| 39 | TOP2A |  |  | 2842 | -0.517 | -0.5101 | Yes |
| 40 | E2F8 |  |  | 2847 | -0.519 | -0.5011 | Yes |
| 41 | CKAP2L |  |  | 2857 | -0.523 | -0.4935 | Yes |
| 42 | ECT2 |  |  | 2858 | -0.523 | -0.4832 | Yes |
| 43 | HJURP |  |  | 2869 | -0.527 | -0.4758 | Yes |
| 44 | ANP32E |  |  | 2877 | -0.530 | -0.4675 | Yes |
| 45 | PSRC1 |  |  | 2944 | -0.569 | -0.4756 | Yes |
| 46 | TACC3 |  |  | 2956 | -0.575 | -0.4675 | Yes |
| 47 | KIF23 |  |  | 2961 | -0.579 | -0.4574 | Yes |
| 48 | TPX2 |  |  | 3006 | -0.606 | -0.4584 | Yes |
| 49 | AURKB |  |  | 3056 | -0.639 | -0.4601 | Yes |
| 50 | CDC45 |  |  | 3081 | -0.661 | -0.4542 | Yes |
| 51 | GTSE1 |  |  | 3083 | -0.663 | -0.4415 | Yes |
| 52 | TMPO |  |  | 3088 | -0.666 | -0.4296 | Yes |
| 53 | NASP |  |  | 3106 | -0.682 | -0.4212 | Yes |
| 54 | POLD3 |  |  | 3122 | -0.695 | -0.4120 | Yes |
| 55 | SMC4 |  |  | 3126 | -0.699 | -0.3992 | Yes |
| 56 | MCM5 |  |  | 3132 | -0.706 | -0.3868 | Yes |
| 57 | GMNN |  |  | 3140 | -0.719 | -0.3748 | Yes |
| 58 | CDK1 |  |  | 3149 | -0.727 | -0.3629 | Yes |
| 59 | MKI67 |  |  | 3158 | -0.738 | -0.3508 | Yes |
| 60 | PRIM1 |  |  | 3160 | -0.739 | -0.3366 | Yes |
| 61 | USP1 |  |  | 3183 | -0.762 | -0.3281 | Yes |
| 62 | CDCA2 |  |  | 3189 | -0.767 | -0.3146 | Yes |
| 63 | CDCA7 |  |  | 3203 | -0.785 | -0.3030 | Yes |
| 64 | CDC6 |  |  | 3204 | -0.785 | -0.2876 | Yes |
| 65 | BIRC5 |  |  | 3208 | -0.793 | -0.2730 | Yes |
| 66 | RRM1 |  |  | 3215 | -0.801 | -0.2591 | Yes |
| 67 | UBE2C |  |  | 3225 | -0.812 | -0.2458 | Yes |
| 68 | NUSAP1 |  |  | 3237 | -0.825 | -0.2329 | Yes |
| 69 | RFC2 |  |  | 3245 | -0.839 | -0.2185 | Yes |
| 70 | DTL |  |  | 3249 | -0.848 | -0.2028 | Yes |
| 71 | CCNE2 |  |  | 3276 | -0.889 | -0.1929 | Yes |
| 72 | FEN1 |  |  | 3278 | -0.890 | -0.1758 | Yes |
| 73 | GAS2L3 |  |  | 3283 | -0.899 | -0.1594 | Yes |
| 74 | HELLS |  |  | 3284 | -0.901 | -0.1417 | Yes |
| 75 | PCNA |  |  | 3319 | -0.963 | -0.1328 | Yes |
| 76 | MCM4 |  |  | 3341 | -1.006 | -0.1192 | Yes |
| 77 | RAD51AP1 |  |  | 3345 | -1.030 | -0.1000 | Yes |
| 78 | GINS2 |  |  | 3386 | -1.156 | -0.0890 | Yes |
| 79 | HMGB2 |  |  | 3390 | -1.184 | -0.0667 | Yes |
| 80 | CLSPN |  |  | 3391 | -1.184 | -0.0435 | Yes |
| 81 | ATAD2 |  |  | 3415 | -1.278 | -0.0252 | Yes |
| 82 | RRM2 |  |  | 3420 | -1.297 | -0.0010 | Yes |
| 83 | TYMS |  |  | 3436 | -1.391 | 0.0219 | Yes |
Table: GSEA details [plain text format]

  

Fig 2: SEURAT\_CELLCYCLE: Random ES distribution      
 Gene set null distribution of ES for **SEURAT\_CELLCYCLE**
